# Clinical, etiological and epidemiological investigations of hand, foot and mouth disease in southern Vietnam during 2015 – 2018

**DOI:** 10.1371/journal.pntd.0008544

**Published:** 2020-08-17

**Authors:** Le Nguyen Thanh Nhan, Truong Huu Khanh, Nguyen Thi Thu Hong, Hoang Minh Tu Van, Le Nguyen Truc Nhu, Nguyen Thi Han Ny, Lam Anh Nguyet, Tran Tan Thanh, Nguyen To Anh, Vu Thi Ty Hang, Phan Tu Qui, Ho Lu Viet, Trinh Huu Tung, Do Quang Ha, Ha Manh Tuan, Guy Thwaites, Nguyen Van Vinh Chau, Louise Thwaites, Nguyen Thanh Hung, H. Rogier van Doorn, Le Van Tan

**Affiliations:** 1 Children’s Hospital 1, Ho Chi Minh City, Vietnam; 2 Oxford University Clinical Research Unit, Ho Chi Minh City, Vietnam; 3 Hospital for Tropical Diseases, Ho Chi Minh City, Vietnam; 4 Children’s Hospital 2, Ho Chi Minh City, Vietnam; 5 University of Medicine and Pharmacy, Ho Chi Minh City, Vietnam; 6 Centre for Tropical Medicine and Global Health, Nuffield Department of Medicine, University of Oxford, Oxford, United Kingdom; The University of Hong Kong, CHINA

## Abstract

Hand, foot and mouth disease (HFMD) continues to challenge Asia with pandemic potential. In Vietnam, there have been two major outbreaks occurring during 2011–2012 (>200,000 hospitalizations and >200 deaths) and more recently in 2018 (>130,000 hospitalizations and 17 deaths). Given the high burden and the complex epidemic dynamics of HFMD, synthesizing its clinical and epidemiological data remains essential to inform the development of appropriate interventions and design public health measures. We report the results of a hospital-based study conducted during 2015–2018, covering the severe HFMD outbreak recently documented in Vietnam in 2018. The study was conducted at three major hospitals responsible for receiving HFMD patients from southern Vietnam with a population of over 40 million. A total of 19 enterovirus serotypes were detected in 1196 HFMD patients enrolled in the clinical study during 2015–2018, with enterovirus A71 (EV-A71), coxsackievirus A6 (CV-A6), CV-A10 and CV-A16 being the major causes. Despite the emergence of coxsackieviruses, EV-A71 remains the leading cause of severe HFMD in Vietnam. EV-A71 was consistently detected at a higher frequency during the second half of the years. The emergence of EV-A71 subgenogroup C4 in late 2018 was preceded by its low activity during 2017–early 2018. Compared with EV-A71 subgenogroup B5, C4 was more likely to be associated with severe HFMD, representing the first report demonstrating the difference in clinical severity between subgenogroup C4 and B5, the two predominant EV-A71 subgenogroups causing HFMD worldwide. Our data have provided significant insights into important aspects of HFMD over four years (2015–2018) in Vietnam, and emphasize active surveillance for pathogen circulation remains essential to inform the local public health authorities in the development of appropriate intervention strategies to reduce the burden of this emerging infections. Multivalent vaccines are urgently needed to control HFMD.

## Author summary

Hand, foot and mouth disease (HFMD) continues to challenge Asia with pandemic potential. Synthesizing epidemiological data remains essential to inform the development of appropriate interventions. We report the results of a comprehensive hospital-based study conducted in southern Vietnam during 2015–2018. A total of 19 different viruses were detected in 1196 HFMD patients enrolled in the study, with enterovirus A71 (EV-A71), coxsackievirus A6 (CV-A6), CV-A10 and CV-A16 being the major causes. EV-A71 was associated with severe HFMD and consistently detected at a higher frequency during the second half of the years. The emergence of EV-A71 subgenogroup C4 in late 2018 was preceded by its low activity during 2017–early 2018. Compared with subgenogroup B5, C4 was more likely to be associated with severe HFMD. Collectively, despite the emergence of coxsackieviruses, EV-A71 remains the major cause of severe HFMD, and multivalent vaccines are urgently needed to control the ongoing HFMD challenge.

## Introduction

Since 1998, hand, foot and mouth disease (HFMD) has emerged, becoming a major public health concern across the Asia-Pacific region pandemic potential. While annually the disease is responsible for over two million hospitalizations in Asia [1–4], sporadic outbreaks have occurred in Europe [5] and in the U.S. in recent years [6, 7]. In Vietnam, since 2011 the average incidence of HFMD is at around 80,000 cases per year, with major outbreaks occurring during 2011–2012 (>200,000 hospitalizations and >200 deaths) and more recently in 2018 (>130,000 hospitalizations and 17 deaths) [8].

HFMD is caused by members of the *Enterovirus* genus, of which enterovirus A71 (EV-A71), coxsackievirus A16 (CV-A16) have historically been regarded as the two most common causes. However, coxsackievirus A6 (CV-A6) and coxsackievirus A10 (CV-A10) have emerged in recent years [9, 10]. As such, these four serotypes have now become predominant causes of HFMD worldwide. Additionally, the epidemic dynamics of HFMD in Asia have strongly been driven by the emergence of these different serotypes, especially EV-A71, which is historically itself divided into major genogroups, including A, B and C, and subgenogroups: B1 –B5 and C1 –C5, accordingly [11]. In recent years, newly described genogroups of EV-A71 have been reported from India (genogroups D and G) [12, 13], Central Africa, and Madagascar (genogroup E and F) [14].

HFMD mainly affects children of 5 years old or less and is clinically a mild disease, usually resolving within a week of onset. Severe clinical complications, however, may occur, which are often associated with EV-A71 and can be fatal [15]. Despite the public health significance of HFMD, there are no clinically proven effective antivirals to offer the affected patients, while vaccine development (including monovalent and multivalent) is still ongoing [16, 17]. Notably, inactivated EV-A71 vaccines have recently been successfully developed in China [18–20], although the use of these EV-A71 vaccines has been restricted to Mainland China.

Given the high endemicity and the complex epidemic dynamics of HFMD, synthesizing clinical, epidemiology and etiology of this emerging infection remains essential to inform the development of appropriate interventions (including vaccines) and design public health measures in order to reduce its global burden. As such since 2013, we have been running a comprehensive hospital based study at three major hospitals in Ho Chi Minh City aiming at capturing long-term data on these aspects of this emerging infection [9, 21–23]. Herein, we report the results of our investigations for the period during 2015–2018, covering the most recent severe HFMD outbreak recently documented in Vietnam [8].

## Materials and methods

### Ethics statement

The Institutional Review Board of CH1, CH2 and HTD, and the Oxford Tropical Research Ethics Committee (OxTREC) approved the study. Written informed consent was obtained from a parent or guardian of each enrolled patients.

### Settings

The clinical and patient data used in the present study were derived from an ongoing clinical study of HFMD, which has been conducted at the Hospital for Tropical Diseases (HTD), Children’s Hospital 1 (CH1) and Children’s Hospital 2 (CH2) in Ho Chi Minh City, Vietnam since 2013 [8, 23]. These are tertiary and referral hospitals receiving HFMD patients, especially cases with severe diseases from Ho Chi Minh City and southern provinces in Vietnam with a population of over 40 million.

The aims the present study were to describe the etiological agents associated with HFMD occurring southern Vietnam between July 2013 and December 2018, and to compare the associated epidemiological and clinical characteristics of HFMD in patients infected by different pathogens. The results of the first part of the clinical study for the period from July 2013 to July 2015 have previously been reported elsewhere [23]. Here, we focus our analysis for the period from August 2015 to December 2018.

### Patient enrollment and data collection

We screened any patient ≤12 years of age presenting to outpatient departments or admitted to inpatient wards of the 3 participating hospitals with a clinical diagnosis of HFMD and, if outpatients, an illness day of ≤3 days for enrolment in our study. We excluded any patient in whom the attending physician believed another diagnosis was more likely. Because of the waning of the epidemic in 2017 and the availability of the resources, from January to December 2018, our recruitment focus was reduced to CH1.

We collected information regarding demographic, clinical signs/symptoms (including clinical grades) on enrolment to the study and (if inpatients) daily until discharge or day seven of hospitalization (whichever came first), treatments, laboratory tests, length of hospital stay (inpatients only), and outcomes. Additionally, we sampled acute throat- and rectal swabs at enrolment from each participant.

### HFMD clinical grade classification

According to the Vietnamese Ministry of Health, HFMD is clinically divided into four major grades: Grade 1 is assigned to patients presenting with mouth ulcers or vesicles/papules on hands, feet or buttocks, with or without mild fever (<39°C); Grade 2 is further divided into Grade 2A (central nervous system (CNS) involvement, (myoclonus reported by parents or caregivers only, fever >39°C or ataxia)), Grade 2B1 (myoclonus observed by medical staff or history of myoclonus and lethargy or pulse higher than 130 bpm), and Grade 2B2 (ataxia, cranial nerve palsies, limb weakness, nystagmus, persistent high fever or pulse higher than 150 bpm); Grade 3 involves autonomic dysfunction with sweating, hypertension, tachycardia and tachypnea and Grade 4 is for disease with additional cardio-pulmonary compromise with pulmonary edema or shock syndrome [3, 24]. Patients with Grade 2B1 or above are considered to have severe HFMD due to presumed involvement of the central nervous system, and often require intravenous immunoglobulin administration according to local guidelines. This classification is in line with the clinical classification of the WHO, for which Grade 1, Grade 3 and Grade 4 of the Vietnamese systems are correspondingly similar to uncomplicated HFMD, HFMD with ANS dysregulation and HFMD with cardiopulmonary failure, respectively [24]. However, according to the Vietnamese classification system, HFMD with CNS involvement defined by the WHO, is divided into grade 2A, grade 2B1 and grade 2B2 as above.

### Determination of enterovirus serotype and EV-A71 subgenogroup

**Fig 1:**
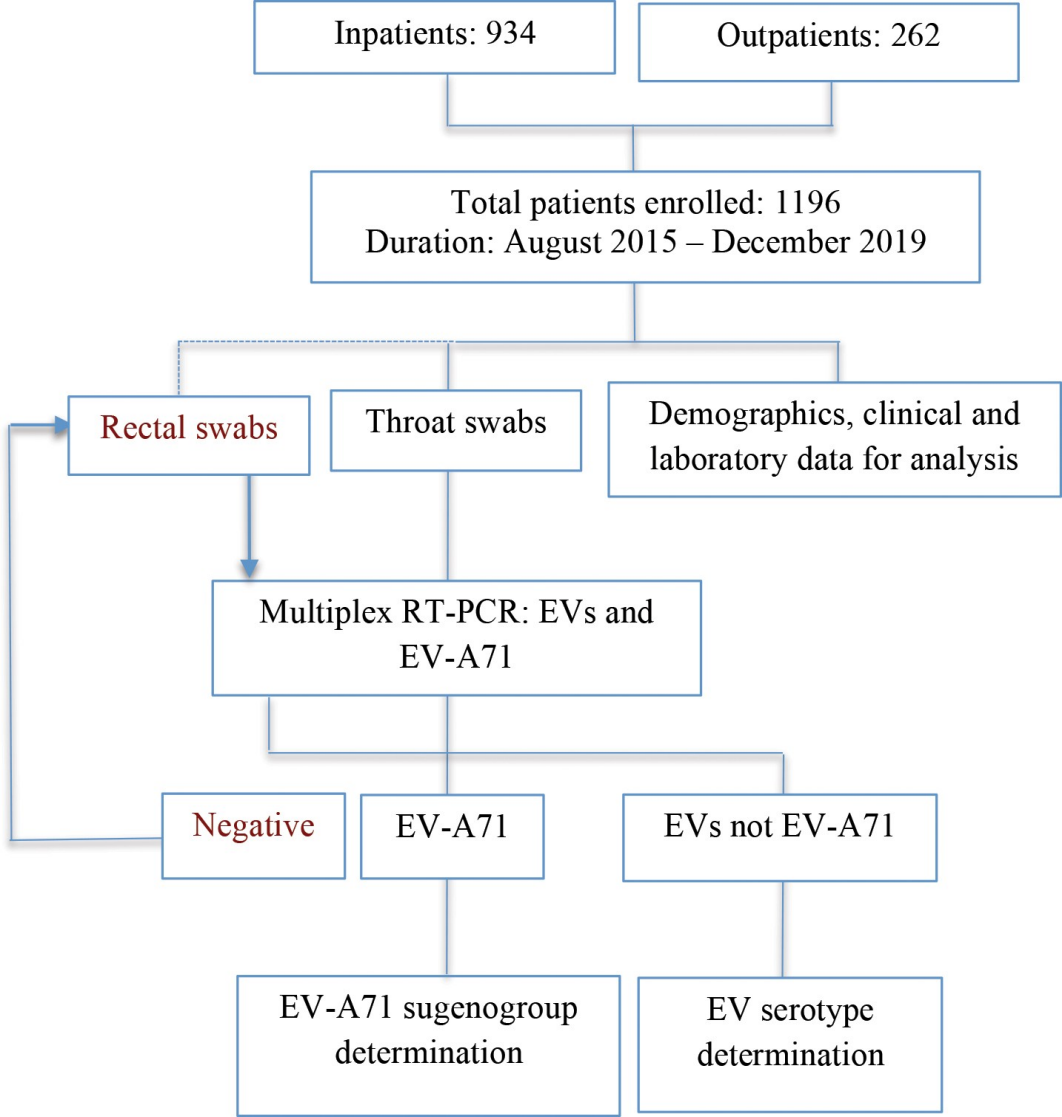
Flowchart illustrating the enrolled in/outpatients and the diagnostic procedure.

Enterovirus diagnosis and serotypes/genogroup determination was carried out using a combination of PCR and sequencing approaches (Fig 1) [25–27]. In brief, we first extract viral RNA from throat swabs collected from of the study participants, and then utilized a one-step multiplex real-time RT-PCR assay to simultaneously detect enteroviruses and EV-A71 in the extracted NA materials [25]. All specimens positive for enterovirus serotype or EV-A71 were then tested to further identify specific enterovirus serotypes or EV-A71 subgenogroups, respectively, using a combination of VP1 PCR amplification and sequencing of the obtained PCR amplicon [3, 26, 27]. Finally, the obtained VP1 sequences were then analyzed using a previously described online tool to determine enterovirus serotype or EV-A71 subgenogroup [28]. In case the one-step multiplex real-time RT-PCR analysis of the throat samples gave a negative result, rectal swabs were further analyzed, and if positive, the same subsequent steps were repeated to identify enterovirus serotypes or EV-A71 subgenogroups [25]. A confirmed diagnosis was established if either throat swab or rectal swab was positive by real time RT-PCR (Fig 1). The diagnostic yield is thus defined as the proportion of patients having a confirmed diagnosis.

### Phenotypic comparison between major subgenogroups of EV-A71

The abovementioned clinical study has been sampling HFMD cases since July 2013. As such, this gave us a unique opportunity to capture the transition between predominant subgenogroups of EV-A71 in Vietnam over a period of 6 years (2013–2018). To assess the differences in clinical phenotypes between subgenogroups of EV-A71, we used a combined dataset of the first- and the second phases of the clinical study (i.e. during July 2013-July 2015 and August 2015-December 2018, respectively).

### Statistical analysis

Data was analyzed using IBM Statistical Package for Social Sciences (SPSS) Version 23.0 (Armonk, NY: IBM Corp.). Chi-square, Fisher exact and Kruskal-Wallis tests were used to compare data between groups of patients when appropriate.

## Results

### Baseline characteristics of the patients

During August 2015 –December 2018, we recruited a total of 1196 children presenting with clinically suspected HFMD. The majority of the participants were young children (median age 18.7 months (range 2.4–118.2)), and predominantly males (n = 746, 62.4%). Nearly half (n = 568, 47.5%) of the patients were from Ho Chi Minh City. In terms of clinical phenotypes, 281 (23.5%) patients had severe HFMD, comprised of 71 cases with grade 2B1, 68 cases with grade 2B2, 136 cases with grade 3 and six cases with grade 4. Milrinone, magnesium sulfate and intravenous immunoglobulin were given to 69 (5.8%), 1 (0.1%) and 215 (18.0%) patients, respectively (Table 1). At discharge, unfavorable outcomes were recorded in 15 patients (1.25%), including three deaths (0.25%) and 12 with complications or underlying diseases (1%) (Table 1). Additional details about the study participants are presented in Table 1.

**Table 1:** Baseline characteristics of the enrolled patients.

Demographics	Total (N = 1196)	Outpatients, N = 262	Inpatients, N = 934	P value	Mild*, N = 915	Severe, N = 281	P value
Median age in month (range)	18.68 (2.40–118.20)	20.62 (4.07–111.13)	18.13 (2.40–118.20)	0.008	17.77 (2.40–111.13)	21.50 (4.07–118.20)	<0.001^
male/female	746/450	170/92	576/358	0.345	564/351	182/99	0.361
HCMC origin (n, %)	568 (47.5)	162 (61.8)	406 (43.5)	<0.001	448 (49.0)	120 (42.7)	0.076
Day from onset to enrolment	1 (0–10)	1 (0–3)	1 (0–10)	0.167	1 (0–10)	2 (0–7)	<0.001^
Length of stay in the hospitals (days)	4 (1–31)	-	4 (1–31)	-	3 (1–31)	5 (1–26)	<0.001^
Environmental factors							
Attend day care centers or school (n, %)	276 (23.1)	97 (37.0)	179 (19.2)	<0.001	206 (22.5)	70 (24.9)	0.404
Number of siblings (median, range)	1 (0–13)	1 (0–6)	0 (0–13)	0.002^	1 (0–6)	0 (0–13)	0.265^
Exposure to HFMD patients (n, %)	150 (12.5)	52 (19.8)	98 (10.5)	<0.001	100 (10.9)	50 (18.0)	0.002
**Clinical features** (n, %)							
Mouth ulcers	1062 (88.8)	214 (81.7)	848 (90.8)	<0.001	819 (89.5)	243 (86.5)	0.159
Fever	936 (78.3)	110 (42.0)	826 (88.4)	<0.001	660 (72.1)	276 (98.2)	<0.001
Skin lesions	1034 (86.5)	234 (89.3)	800 (85.7)	0.126	777 (84.9)	257 (91.5)	0.005
vesicles	313 (26.2)	73 (27.9)	240 (25.7)	0.481	215 (23.5)	98 (34.9)	<0.001
papular	373 (31.2)	109 (41.6)	264 (28.3)	<0.001	308 (33.7)	65 (23.1)	0.001
mix	348 (29.1)	52 (19.8)	296 (31.7)	<0.001	254 (27.8)	94 (33.5)	0.066
Cough	291 (24.3)	82 (31.3)	209 (22.4)	0.003	228 (24.9)	63 (22.4)	0.393
Runny nose	237 (19.8)	78 (29.8)	159 (17.0)	<0.001	186 (20.4)	51 (18.1)	0.493
Diarrhea	117 (9.8)	10 (3.8)	107 (11.5)	<0.001	85 (9.3)	32 (11.4)	0.308
Drowsiness	263 (22.0)	19 (7.3)	244 (26.1)	<0.001	116 (12.7)	147 (52.3)	<0.001
Sweating	17 (1.4)	2 (0.7)	15 (1.6)	0.391	6 (0.7)	11 (3.9)	<0.001
Vomiting	525 (43.9)	61 (23.3)	464 (49.7)	<0.001	349 (38.1)	176 (62.6)	<0.001
Irritability	357 (29.8)	12 (4.6)	345 (36.9)	<0.001	184 (20.1)	173 (61.6)	<0.001
Myoclonus	568 (47.5)	0	568 (60.8)	<0.001	323 (36.0)	245 (87.2)	<0.001
Lethargy	27 (2.3)	3 (1.1)	24 (2.6)	0.239	7 (0.8)	20 (7.1)	<0.001
Tremor	111 (9.3)	2 (0.8)	109 (11.7)	<0.001	9 (1.0)	102 (36.3)	<0.001
Ataxia	3 (0.3)	0	3 (0.3)	1*	0	3 (1.1)	0.013*
Nystagmus	1 (0.1)	0	1 (0.1)	1*	0	1 (0.4)	0.235*
Limb weakness	11 (0.9)	0	11 (1.2)	0.135*	0	11 (3.9)	<0.001*
Hypertension	69 (5.8)	0	69 (7.4)	<0.001*	0	69 (24.6)	<0.001*
**Results of blood tests, median (range)**							
White blood cell (x1000)	12.3 (2.5–76.0)	11.3 (5.6–26.9)	12.6 (2.5–76.0)	0.001	12.2 (3.9–64)	12.4 (2.5–76.0)	0.559^
Platelet (x1000)	321 (29–849)	325 (29–741)	321 (31–849)	0.421	314 (29–788)	347 (38–849)	<0.001^
Blood glucose (mg/L)	101 (42–223)	121 (42–212)	96 (42–223)	<0.001	102 (42–223)	98 (42–176)	0.028^
C reactive protein (mg/L)	9.8 (0–620)	7.8 (0–79)	11.0 (0–620)	0.025	12.0 (0–620)	3.6 (0–87.5)	<0.001^
**Highest grade** (n, %)							
1	251 (21.0)	249 (95.0)	2 (0.2)	<0.001	257 (27.8)	NA	NA
2A	664 (55.5)	13 (5.0)	651 (69.7)	<0.001	668 (72.2)	NA	NA
2B1	71 (5.9)	NA	71 (7.6)	<0.001	NA	71 (25.3)	NA
2B2	68 (5.7)	NA	68 (7.3)	<0.001	NA	68 (24.2)	NA
3	136 (11.4)	NA	136 (14.6)	<0.001	NA	136 (48.4)	NA
4	6 (0.5)	NA	6 (0.6)	0.349	NA	6 (2.1)	NA
**Treatment** (n, %)							
Milrinon	69 (5.8)	0	69 (7.4)	<0.001*	0	69 (5.8)	<0.001*
Magnesium sulfate	1 (0.1)	0	1 (0.1)	1*	0	1 (0.4)	0.235*
IVIg	215 (18.0)	0	215 (23.0)	<0.001*	0	215 (76.5)	<0.001*
**Outcome** (n, %)							
Full recovery	1181 (98.7)	262 (100)	919 (98.4)	0.052	913 (99.8)	268 (95.4)	<0.001
Recovery with complication or underlying diseases#	12 (1.0)	0	12 (1.3)	0.08	2 (0.2)	10 (3.6)	<0.001
Death	3 (0.25)	0	3 (0.3)	1	0	3 (1.1)	0.013

(^): Mann- Whitney U test

(*): Fisher-exact test; other binary variables: Chi-square test

Notes to Table 1: *: grade 1 and 2A, ^#^limb weakness and squint-eyed (n = 2), limb weakness (n = 5), “slow communication” (n = 1), cranial nerve VII paralysis (n = 1), unconsciousness (n = 1), Guillain Barre syndrome (n = 1), prolonged fever and paralyzed diaphragm (n = 1), severe anemia (n = 2) and subacute encephalitis (n = 1), NA: nonapplicable

Of 1196 patients enrolled in the study, there were a total of 262 outpatients (21.9%) and 934 inpatients (78.1%). There were considerable differences in demographics, clinical presentation and results of blood biochemistry parameters between out- and inpatients (Table 1). Notably, inpatients were younger than outpatients and were more likely to come from other provinces (Table 1). Inpatients had a slightly higher while blood cell count and C reactive protein, but a lower level of blood glucose (Table 1).

Compared with mild patients (Grade 1 or 2A), severe patients (Grade 2B1 or above) were admitted to the hospital later. Additionally, severe patients were slightly older, had a higher platelet count, but a lower level blood glucose and C reactive protein level than those with mild disease. Otherwise, the differences between out-and inpatients as well as between mild and severe patients in clinical presentations merely reflect the differences in clinical criteria utilized to classify HFMD grades.

Between August 2015 and December 2017 when the study was conducted at the three participating hospitals, 785 patients (785/1196, 66%) were enrolled. There were considerable differences in demographics, environmental factors (attending day care centers, exposure to HFMD cases) and clinical characteristics between patients enrolled at CH1 and those enrolled at CH2 and HTD (S1 Table).

### Results of enteroviral investigation: an overview

Using one-step multiplex real-time RT-PCR, we identified evidence of enterovirus in 977 (81.6%) of 1196 study participants, including 339 EV-A71 (28.3%) and 638 non-EV-A71 enteroviruses (53.3%). Of the 339 EV-A71 positive cases, subgenogroup determination was successful in 124 (36.5%) cases (including 52 subgenogroup B5 and 72 subgenogroup C4). Of the non-EV-A71 enterovirus positive cases, sequencing of VP1 fragment was successful in 494/638 (77.4%), which consisted of 18 different enterovirus serotypes, with CV-A6, CV-A10 and CV-A16 being the major serotypes (Fig 1). The frequency of the remaining 15 enterovirus serotypes are presented in S2 Table.

### The frequency of enterovirus serotypes detected in out- and inpatients

**Fig 2:**
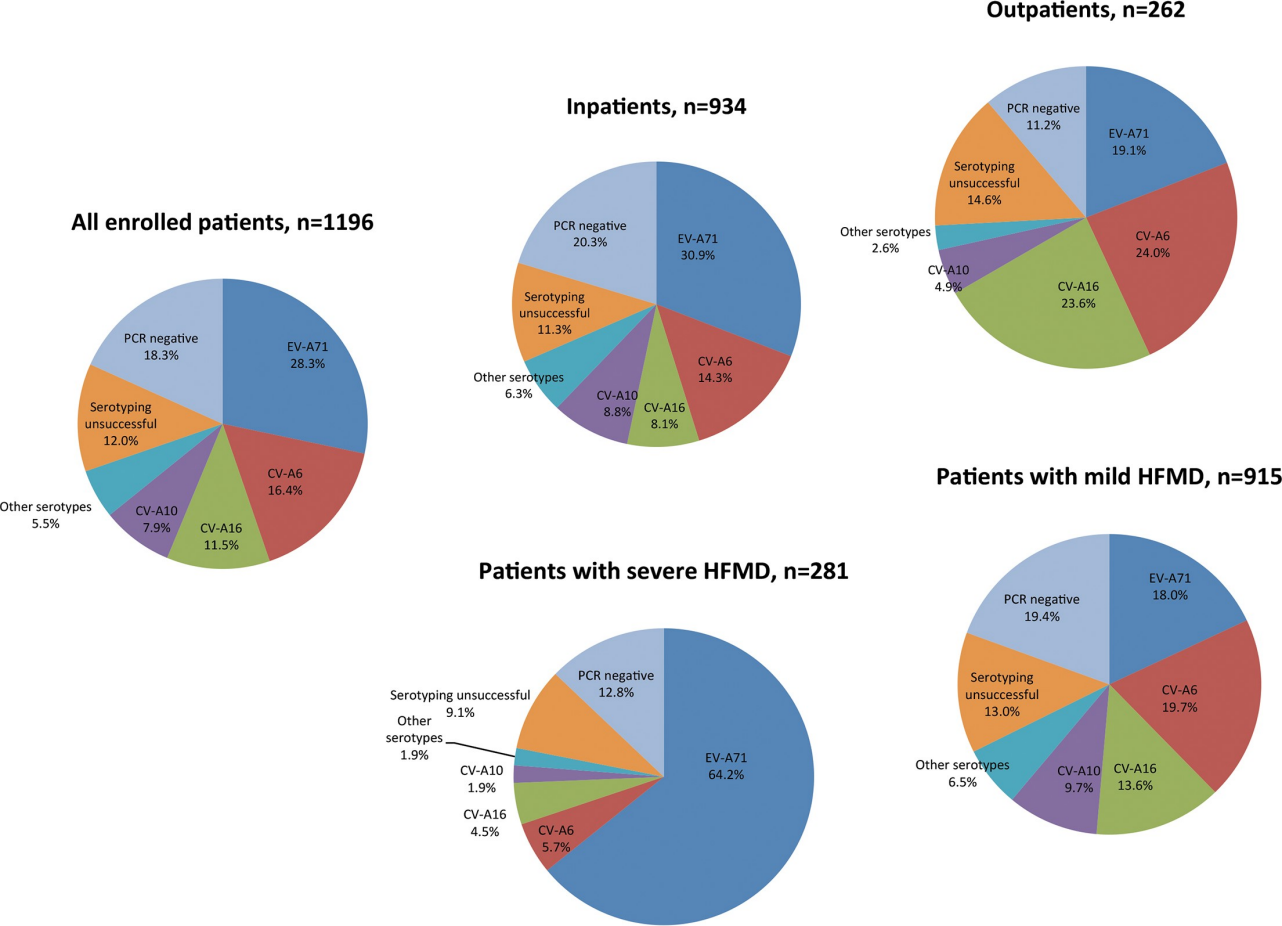
Pie charts showing the detection rates of enterovirus serotypes in all HFMD patients enrolled in the study and groups of patients with severe and mild HFMD, and in- and outpatients.

The overall PCR diagnosis yield of the inpatient group was lower than that of the outpatient group (Fig 1). Likewise, there were some differences in the frequency of the four predominant enterovirus serotypes detected in these two groups, with EV-A71 being the major viruses detected in the inpatient group followed by CV-A6, CV-A10 and CV-A16. In contrast with the inpatient group, CV-A6 and CV-A16 were the two leading viruses detected at comparable frequencies in the outpatient group, followed by EV-A71 and CV-A10 (Fig 2).

### The frequency of enterovirus serotypes detected in patients with mild and severe HFMD

There were considerable differences in the overall diagnostic yield and the detection rates of predominant enterovirus serotypes (EV-A71, CV-A6, CV-A10 and CV-A16) between patients with severe and mild HFMD (Fig 2). More specifically, of the 281 patients presenting with severe HFMD, EV-A71 was the virus detected in 64.2% of the patients, followed by sporadic detections of CV-A6 (5.7%), CV-A16 (4.5%) and CV-A10 (1.9%) and other serotypes (1.9%) (Fig 2 and S2 Table). Meanwhile, of the 915 patients with mild HFMD, the detections were comparable between EV-A71 (18%) and CV-A6 (19.7%), followed by CV-A16 (13.6%) and CV-A10 (9.7%). The frequency of the remaining serotypes detected in out/inpatients and patients with mild/severe HFMD are presented in S2 Table.

### Associated demographics, clinical characteristics and outcome of predominant enterovirus serotypes, and age groups

Detailed comparisons between patients positive for predominant enterovirus serotypes (EV-A71, CV-A6, CV-A10 and CV-A16) are presented in Table 2. Generally, there were similarities between patients infected with CV-A6, CV-A10 and CV-A16, while the clinical sign/symptoms as myoclonus, tremor, hypertension, vomiting and irritability, reflecting the characteristics of severe HFMD, were more often observed in EV-A71 infected patients. Likewise, unfavorable outcomes, especially death, were almost exclusively recorded in patients with EV-A71 infections. Other notable differences included age, the frequency of skin lesions, and blood biochemistry results. More specifically, patients with CV-A6 and CV-A10 infection were younger than those infected with EV-A71 and CV-A16. Skin lesions were recorded in only 61.1% (58/95) of CV-A10 positive patients, but in almost all (>91%) EV-A71/CV-A6/CV-A16 infected patients. Patients with CV-A10 and CV-A6 infection had a higher level of C reactive protein and a slightly lower level of platelet count than EV-A71/CV-A16 infected patients.

**Table 2:** Characteristics of HFMD patients positive for different viruses.

	EV-A71 (N = 340)	CV-A6 (N = 196)	CV-A10 (N = 95)	CV-A16 (N = 137)	P value
**Demographics**					
Median age in month (range)	22.07 (4.17–118.20)	15.50 (4.40–55.40)	15.23 (2.40–77.17)	20.70 (8.97–106.07)	<0.001 (*)
Sex (male/female)	215 /125	142/54	55/40	80/57	0.107
HCMC origin (n, %)	158 (46.5)	91 (46.4)	52/43	73/64	0.023
Day from onset to enrolment	2 (0–7)	1 (0–10)	1 (0–5)	1 (0–5)	<0.001*
Length of stay in the hospitals (days)	4 (1–26)	3 (1–31)	3 (1–8)	3 (1–12)	<0.001*
Environmental factors					
Attend day care centers or school (n, %)	96 (28.2)	33 (16.8)	12 (12.6)	37 (27.0)	0.001
Number of siblings (median, range)	1 (0–6)	0 (0–5)	1 (0–13)	1 (0–5)	0.285*
Exposure to HFMD patients (n, %)	68 (20.0)	17 (8.7)	10 (10.5)	16 (11.7)	0.001
**Clinical features** (n, %)					
Skin lesions	324 (95.3)	182 (92.9)	57 (60.0)	132 (96.4)	<0.001
vesicle	131 (38.5)	35 (17.9)	27 (28.4)	19 (13.9)	<0.001
papular	97 (28.5)	67 (34.2)	18 (18.9)	63 (46.0)	<0.001
mix	96 (28.2)	80 (40.8)	12 (12.6)	50 (36.5)	<0.001
Skin lesions on palms or soles (n, %)	307 (90.3)	173 (88.3)	33 (34.7)	126 (92.0)	<0.001
Skin lesions on knees or elbows (n, %)	192 (56.5)	147 (75.0)	30 (31.6)	113 (82.5)	<0.001
Skin lesions on buttocks (n, %)	248 (72.9)	159 (81.1)	48 (50.5)	108 (78.8)	<0.001
Mouth ulcers (n, %)	295 (86.8)	172 (87.8)	94 (98.9)	131 (95.6)	<0.001
Fever	304 (89.4)	136 (69.4)	75 (78.9)	78 (56.9)	<0.001
Cough	76 (22.4)	39 (19.9)	26 (27.4)	31 (22.6)	0.560
Runny nose	54 (15.9)	32 (16.3)	22 (23.2)	36 (26.3)	0.032
Diarrhea	25 (7.4)	10 (5.1)	11 (11.6)	11 (8.0)	0.263
Drowsiness	143 (42.1)	22 (11.2)	9 (9.5)	8 (5.8)	<0.001
Sweating	8 (2.4)	4 (2.0)	1 (1.1)	2 (1.5)	0.833
Vomiting	175 (51.5)	85 (43.4)	30 (31.6)	43 (31.4)	<0.001
Irritability	164 (48.2)	38 (19.4)	11 (11.6)	24 (17.5)	<0.001
Myoclonus	233 (68.5)	64 (32.7)	33 (34.7)	38 (27.7)	<0.001
Lethargy	17 (5.0)	2 (1.0)	2 (2.1)	1 (0.7)	0.015
Tremor	88 (25.9)	6 (3.1)	3 (3.2)	1 (0.7)	<0.001
Ataxia	3 (0.9)	1 (0.5)	0	0	0.557
Nystagmus	1 (0.3)	0	0	0	0.739
Limb weakness	5 (1.5)	1 (0.5)	0	1 (0.7)	0.484
Hypertension	47 (13.8)	5 (2.6)	1 (1.1)	2 (1.5)	<0.001
**Diagnosis and lab results**					
White blood cells (x1000)	12.5 (3.2–31.8)	12.9 (4.2–76.0)	14.55 (5.0–34.7)	11.6 (2.5–45.5)	<0.001*
Platelet	350.5 (29–844)	324 (35–849)	308.5 (68–590)	324 (74–741)	<0.001*
Blood glucose (mg/L)	100 (42–198)	102 (42–174)	96 (48–212)	104.5 (42–195)	0.596*
C reactive protein (mg/L)	3.25 (0–74)	19.9 (1.0–620)	25.1 (1.0–187.0)	8 (0.4–79.8)	<0.001*
**Highest grade** (n, %)					
1	50 (14.7)	55 (28.1)	14 (14.7)	63 (46.0)	<0.001
2	113 (33.2)	125 (63.8)	76 (80.0)	60 (43.8)	<0.001
2B1	32 (9.4)	5 (2.6)	3 (3.2)	8 (5.8)	0.007
2B2	50 (14.7)	4 (2.0)	0	1 (0.7)	<0.001
3	89 (26.2)	7 (3.6)	2 (2.1)	5 (3.6)	<0.001
4	6 (1.8)	0	0	0	0.055
**Treatments** (n, %)					
Milrinon	49 (14.4)	4 (2.0)	1 (1.1)	2 (1.5)	<0.001
Magnesium sulfate	1 (0.3)	0	0	0	0.285
IVIg	135 (15.6)	10 (5.1)	2 (2.1)	6 (4.4)	<0.001
**Outcome** (n, %)					
Full recovery	331 (97.4)	195 (99.5)	95 (100)	135 (98.5)	0.137
Recovery with complication	7 (2.1)	1 (0.5)	0	2 (1.5)	0.290
Death	2 (0.6)	0	0	0	0.417

**Note to Table 2**: *Kruskal-Wallis test; Binary variables: Chi-Square test

Overall, infants and children presented with similar clinical characteristics, and had comparable laboratory findings and outcome (S3 Table). Otherwise, the remaining differences were likely attributed to the predominance of EV-A71 in children.

### Temporal distribution of predominant enterovirus serotypes

**Fig 3:**
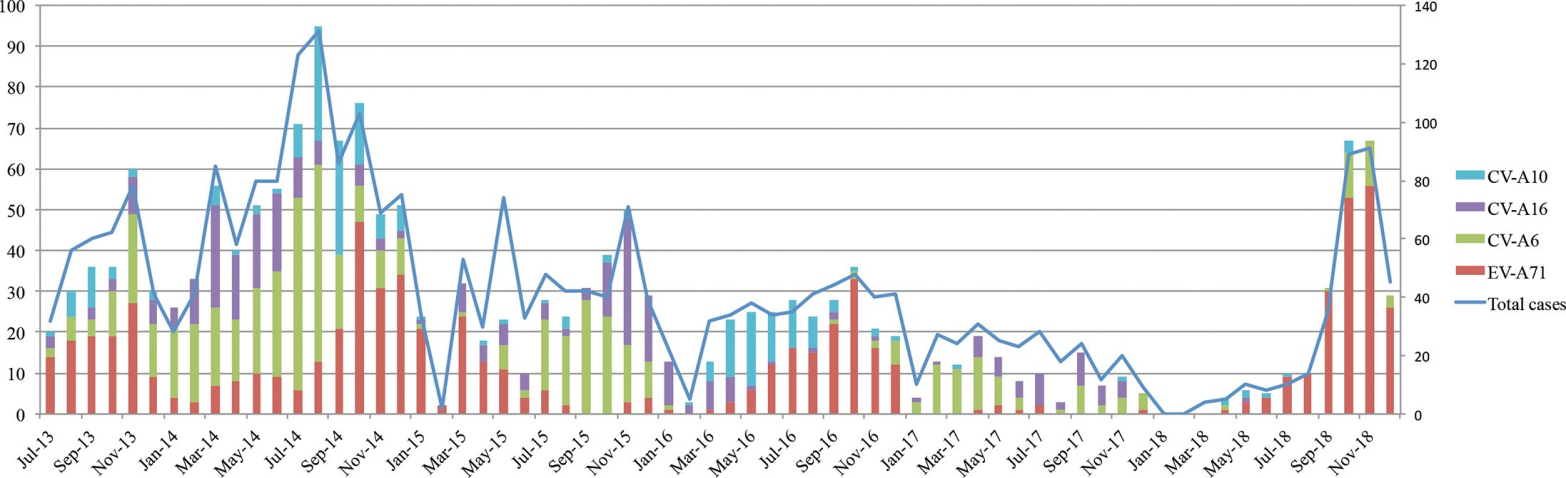
Temporal distribution of four major enterovirus serotypes during the study period and the period from July 2013 to July 2015. **Note to Fig 3:** The left Y-axis shows the number of cases of specific serotypes. The right Y-axis shows the number of total cases. The synthesized data also covers the period from July-2013 to July 2015 which was previously reported.

Temporally, there were fluctuations in the detection rates of predominant enterovirus serotypes (EV-A71, CV-A6, CV-A10 and CV-A16) over the study period. Generally, EV-A71 activity was consistently high during the second half of the years, while CV-A6, CV-A10 and CV-A16 predominantly circulated in the first half of the years (Fig 3). Of particular note also was the almost absence of EV-A71 during 2017 and the first half of 2018 followed by its emergence in late 2018.

### Temporal distribution of EV-A71 subgenogroup C4 and B5 and their clinical comparison

**Fig 4:**
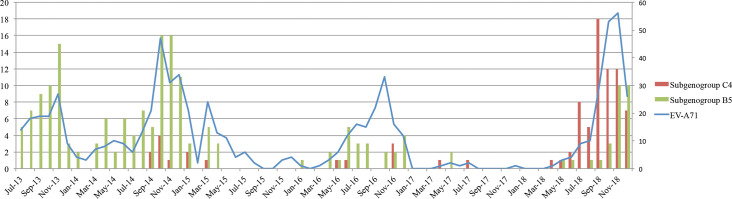
Reconstructed temporal distribution of EV-A71 subgenogroup C4 and B5 during 2013–2018. **Notes to Fig 4:** The left Y-axis shows the number of subgenogroup C4/B5 cases. The right Y-axis shows the number of EV-A71 cases. Data were synthesized for the period during July 2015 and December 2018 which was also derived from our previous reports for the period from July 2013 to July 2015.

Together with the previous phase of the study [21, 23], information about EV-A71 subgenogroups was available in a total of 273 patients (including 190 subgenogroup B5 and 83 subgenogroup C4). Temporally, these two subgenogroups replaced each other over six years in southern Vietnam (Fig 4). Specifically, from July 2013 to December 2016, B5 was dominant. After a period of low EV-A71 activity in 2017 and early 2018, C4 became the major virus detected between July and October 2018, followed by co-circulation of C4 and B5 during the last two months of 2018.

Clinically, patients infected with subgenogroup C4 were more likely to have severe HFMD than those infected with subgenogroup B5. The results were consistent for the whole study (Table 3). In 2018, more severe patients were also recorded for subgenogroup B5. Notably, during this period, B5 infected patients was younger than those recorded in previous years.

**Table 3:** Phenotypic comparison between EV-A71 subgenogroup C4 and B5.

	2013–2017			2018			The whole study period (2013–2018)		
	C4, N = 17	B5, N = 164	P value, (OR; 95%CI)	C4, N = 66	B5, N = 26	P value, (OR; 95%CI)	C4, N = 83	B5, N = 190	P value, (OR; 95%CI)
**Median of age in months (range)**	23.03 (13.97–38.40)	20.15 (5–110)	P = 0.381 *	22.50 (8.77–65.87)	14.68 (4.17–118.20)	P = 0.009*	23.03 (8.77–65.87)	18.42 (4.17–118.20)	P = 0.022*
**Severe HFMD, n (%)**	12 (70.6)	25 (15.2)	<0.001 (13.3; 4.3.–41.2)	51 (77.3)	14 (53.8)	0.026 (2.9; 1.1–7.6)	63 (76)	39 (21)	<0.001 (12.2; 6.6–22.5)
2B1	4	12		4	1		8	13	
2B2	3	9		12	4		15	13	
3	5	4		32	9		37	13	
4	0	0		3	0		3	0	

**Notes to Table 3**: mild: grade 1 or 2A, severe: grade 2B1 or above, *comparison between mild and severe groups *Mann-Whitney U test

## Discussion

Here we report the results of clinical, etiological and epidemiological investigations of children presenting with clinically suspected HFMD. Study participants were admitted to or sought for medical treatment at outpatient departments at major hospitals in Ho Chi Minh City Vietnam, where the majority of HFMD cases in Vietnam has been reported to date. Together with our previous reports [8, 23], our data have thus provided significant insights into important aspects of this emerging infection over six years (2013–2018). More specifically, we have demonstrated the emergence CV-A6 as a major cause of HFMD in Vietnam, consistent with the situation worldwide [6, 9]. Yet, despite the emergence of CV-A6 and to some extent CV-A10, EV-A71 remains the leading cause of severe HFMD in Vietnam. Additionally, the results expanded our knowledge about the predominant EV-A71 subgenogroups responsible for the 2018 outbreak; subgenogroup C4 was the major virus circulating in Vietnam between July and October 2018 [8], followed by the co-circulation of both C4 and B5 in the last two months of the 2018 outbreak. Notably, however, in contrast to previous years, during the 2018 outbreak B5 seemed to attack younger children. This may in turn explain an increase in the proportion of B5 patients with severe HFMD in 2018.

The underlying mechanisms behind the temporal changes in HFMD viruses during the study remains to be determined [23, 29]. Notably, in contrast to the continuous circulation of EV-A71 during the first two years of the study (2013–2015) [23], results of the present study show that the emergence of EV-A71 during the second half 2018 was preceded by the low detection rate of EV-A71 in HFMD cases enrolled in 2017 and early 2018. This suggests that EV-A71 endemicity in Vietnam was low during this period, which may have resulted in the accumulation of a sufficient number of susceptible young children leading to the emergence of EV-A71 in late 2018 [8, 30]. Collectively, the data suggest that active surveillance for pathogen circulation, especially viruses that are known to cause severe outbreaks, e.g. EV-A71, remains essential to inform the local public health authorities in response to HFMD outbreaks and the development of appropriate/effective HFMD vaccines.

Our data have demonstrated that, compared with subgenogroup B5, C4 was more likely to cause severe HFMD, suggesting that their virulence may differ. Viruses of subgenogroups C and B, especially C4 and B5, have been predominantly detected in recent outbreaks in the Asia-Pacific region, including China, Taiwan and Vietnam, with frequent switches in predominant subgenogroups overtime. To this end, existing data fails to link the association between specific (sub)genogroups and severe HFMD. In Vietnam, historically subgenogroup C4 has been linked with major outbreaks during 2011–2012 [3] and most recently 2018 [8]. Additionally, data of the present study show that C4 infected patients were more likely to have severe HFMD than B5 patients. As such, the results parallel observational data from Thailand and China, where C4 has been responsible for major HFMD outbreaks [1, 31]. In contrast, elsewhere in Taiwan, subgenogroup C2 and B4 were responsible for the massive outbreak involving 1.5 million infections and 78 death in 1998 [32], while C4 and B5 have both been linked with recent outbreaks [33, 34]. Collectively, while our data represent the first report demonstrating the difference in clinical severity between subgenogroup C4 and B5, further research should address the underlying factors that determine the scales and the severity of HFMD in specific localities, paramount importance for development of intervention strategies.

HFMD is generally a mild infection, with severe disease occurring in only a small proportion of the affected patients. It remains unresolved regarding the factors determining the clinical consequences of the affected individuals, although virologically EV-A71 is often associated with severe HFMD. Notably, late hospital admission and older children were more often recorded among EV-A71 infected patients and among patients presenting with severe HFMD, suggesting that the time since onset of illness to hospital admission and age may play a role.

Our overall PCR diagnostic yield of 81.6% is within the ranges of previous reports [1, 23, 35]. The failure to detect enterovirus in the remaining 18.4% of the patients may have been attributed to the low level of viral load in the tested samples and/or the miss-inclusion of patients with other diseases presenting with rash (e.g. measles or rubella) into the study. The higher PCR detection rate of the outpatient group as compared to that of the inpatient group may be attributed to the longer duration of illness at enrolment of the inpatients, albeit not statistically significant. Likewise, because molecular typing assay is a conventional RT-PCR based approach, which is less sensitive than real time RT-PCR [25], we were not able to identify specific enterovirus serotypes in 12.6% of the patients with a confirmed EVs diagnosis. However, similar to previous reports [35, 36], the results show that HFMD is diverse in etiology with over 19 enterovirus serotypes being linked with the current HFMD epidemic in Vietnam. Given the weak/lack of cross-neutralization between them, especially among predominant serotypes [17, 30, 37, 38], the data thus indicate that multivalent vaccines will be needed to control this ongoing epidemic. Although challenging to develop, multivalent vaccines are urgently required [35, 39, 40], especially given the high burden of HFMD [22, 41, 42]. EV-A71 vaccines have been successfully developed elsewhere [18, 20, 38] and could potentially reduce the major impact of HFMD in the region.

The major strength of our study is that it was based at tertiary referral hospitals in Chi Minh City, which are responsible for receiving HFMD from southern Vietnam with a population of over 40 million. However, patients with mild HFMD often seek for medical treatments at local hospitals or private clinics, or being managed at home by parents. Additionally, we were not able to expand our sampling to the central and northern Vietnam and the epidemic picture in these respective areas remains unclear. An additional limitation includes our adjustment in sampling approach from three to one study site (CH1) in 2018. Collectively, our data may not well be generalizable for the ongoing epidemic patterns of HFMD in Vietnam during the study period.

To summarize, our results showed the complex dynamics of the ongoing HFMD epidemic in Vietnam, which has been driven in part by the emergence of different viruses, especially EV-A71 and CV-A6. The results indicate that there is an urgent need to develop multivalent vaccines in order to control this emerging infection and that active surveillance for pathogen circulation remains essential to inform the local public health authorities in the development of appropriate intervention strategies to reduce the burden of HFMD.

## Supporting information

S1 STROBE Checklist STROBE checklist. (DOC)

S1 Table Assessing the homogeneity among patients enrolled at CH1 and CH2 and HTD combined. (DOCX)

S2 Table Frequency of other enterovirus serotypes detected in HFMD cases enrolled in the clinical study. (DOCX)

S3 Table Comparing between infants and children. (DOCX)
