# Supplementary material for: Clinical, etiological and epidemiological investigations of hand, foot and mouth disease in southern Vietnam during 2015 – 2018
Source: PLoS Negl Trop Dis. 2020 Aug 17;14(8):e0008544. doi: 10.1371/journal.pntd.0008544 (PMC7451980; doi:10.1371/journal.pntd.0008544)
Supplement: S1 Table — (DOCX) [file pntd.0008544.s002.docx]

**Supplementary Table 1**: Assessing the homogeneity among patients enrolled at CH1 and CH2 and HTD combined

| **Variables** | **Inpatients (n=577)** | | | **Outpatients (n=208)** | | |
| --- | --- | --- | --- | --- | --- | --- |
|  | **CH1 (n=431)** | **CH2&HTD (n=146)** | **P-value** | **CH1 (n=32)** | **CH2&HTD (n=176)** | **P-value** |
| **Demographics** |  |  |  |  |  |  |
| Median age in month (range) | 16.43 (3.20-102.93) | 17.95 (4.07-62.57) | 0.229^ | 17.97 (8.0-60.93) | 21.37 (4.07-113.13) | 0.372^ |
| Male/female | 270/161 | 82/64 | 0.165 | 15/17 | 119/57 | 0.024 |
| HCMC origin (n, %) | 174 (40.4) | 85 (58.2) | <0.001 | 22 (68.8) | 114 (64.8) | 0.664 |
| Day from onset to enrolment | 1 (0-10) | 2 (0-5) | <0.001^ | 1 (0-3) | 1 (0-3) | 0.489^ |
| Length of stay in the hospitals | 3 (1-31) | 5 (1-13) | <0.001^ | - | - |  |
| **Environmental factors** |  |  |  |  |  |  |
| Attend day care centers or school (n, %) | 66 (15.3) | 39 (26.7) | 0.002 | 9 (28.1) | 74 (42.0) | 0.139 |
| Number of siblings (median, range) | 0 (0-13) | 0 (0-6) | 0.705^ | 1 (0-6) | 1 (0-4) | 0.973^ |
| Exposure to HFMD patients (n, %) | 24 (5.6) | 17 (11.6) | 0.014 | 6 (18.8) | 35 (19.9) | 0.882 |
| **Clinical features (n, %)** |  |  |  |  |  |  |
| Mouth ulcers | 400 (92.8) | 127 (87.0) | 0.031 | 29 (90.6) | 162 (92.0) | 0.730* |
| Fever | 343 (79.6) | 130 (89.0) | 0.010 | 11 (34.4) | 61 (34.7) | 0.975 |
| Skin lesions | 376 (87.2) | 89 (61.0) | <0.001 | 24 (75.0) | 161 (91.5) | 0.006 |
| vesicles | 78 (18.1) | 19 (13.0) | 0.156 | 4 (12.5) | 29 (16.5) | 0.793* |
| papular | 160 (37.1) | 26 (17.8) | <0.001 | 4 (12.5) | 96 (54.5) | <0.001 |
| mix | 138 (32.0) | 44 (30.1) | 0.672 | 16 (50.0) | 36 (20.5) | <0.001 |
| Cough | 78 (18.1) | 45 (30.8) | 0.001 | 7 (21.9) | 66 (37.6) | 0.088 |
| Runny nose | 56 (13.0) | 52 (35.6) | <0.001 | 6 (18.8) | 68 (38.6) | 0.031 |
| Diarrhea | 52 (12.1) | 11 (7.5) | 0.127 | 2 (6.3) | 6 (3.4) | 0.356* |
| Drowsiness | 33 (7.7) | 4 (2.7) | 0.048* | 2 (6.3) | 4 (2.3) | 0.231 |
| Sweating | 5 (1.2) | 1 (0.7) | 1* | 0 | 2 (1.1) | 1* |
| Vomiting | 162 (37.6) | 51 (34.9) | 0.566 | 4 (12.5) | 26 (14.8) | 1* |
| Irritability | 45 (10.4) | 10 (6.8) | 0.202 | 2 (6.3) | 0 | 0.023* |
| Myoclonus | 215 (49.9) | 85 (58.2) | 0.081 | 0 | 0 | NA |
| Lethargy | 11 (2.6) | 2 (1.4) | 0.532* | 0 | 3 (1.7) | 1* |
| Tremor | 18 (4.2) | 9 (6.2) | 0.326 | 0 | 2 (1.1) | 1* |
| Ataxia | 0 | 2 (1.4) | 0.064* | 0 | 0 | NA |
| Nystagmus | 0 | 0 | NA | 0 | 0 | NA |
| Limb weakness | 0 | 4 (2.7) | 0.004* | 0 | 0 | NA |
| Hypertension | 10 (2.3) | 4 (2.7) | 0.759* | 0 | 0 | NA |
| **Results of blood tests, median (range)** |  |  |  |  |  |  |
| White blood cell (x1000) | 12.50 (3.20-64.0) | 12.90 (3.30-34.70) | 0.745^ | 9.40 (6.50-16.30) | 11.75 (5.60-26.90) | <0.001^ |
| Platelet (x1000) | 312 (35.50-720) | 308 (31.0-501) | 0.493^ | 317 (186-482) | 326 (28.60-741) | 0.936^ |
| Blood glucose (mg/L) | 91.0 (42.0-181) | 128 (53.0-223) | <0.001^ | 118 (42.0-212) | 121 (61.0-198) | 0.340^ |
| C reactive protein (mg/L) | 15.60 (0-620) | 12.0 (0-70.0) | 0.179^ | 7.80 (0-48.3) | 7.70 (4.0-79.0) | 0.798^ |
| **Pathogens** |  |  |  |  |  |  |
| EV-A71 | 73 (16.9) | 43 (29.5) | 0.001 | 0 | 34 (19.3) | 0.003* |
| CV-A6 | 93 (21.6) | 9 (6.2) | <0.001 | 10 (31.3) | 39 (22.2) | 0.265 |
| CV-A10 | 47 (10.9) | 26 (17.8) | 0.030 | 2 (6.3) | 10 (5.7) | 1* |
| CV-A16 | 40 (9.3) | 10 (6.8) | 0.367 | 9 (28.1) | 53 (30.1) | 0.821 |
| Other EV serotypes | 79 (18.3) | 30 (20.5) | 0.554 | 9 (28.1) | 23 (13.1) | 0.030 |
| PCR negative | 99 (23.0) | 28 (19.2) | 0.339 | 2 (6.3) | 17 (9.7) | 0.744* |
| **Highest grade (n, %)** |  |  |  |  |  |  |
| 1 | 1 (0.2) | 1 (0.7) | 0.442 | 31 (96.9) | 175 (99.4) | 0.285* |
| 2A | 378 (87.7) | 89 (61.0) | <0.001 | 1 (3.1) | 1 (0.6) | 0.285* |
| 2B1 | 17 (3.9) | 34 (23.3) | <0.001 | 0 | 0 | NA |
| 2B2 | 10 (2.3) | 10 (6.8) | 0.010 | 0 | 0 | NA |
| 3 | 25 (5.9) | 11 (7.5) | 0.454 | 0 | 0 | NA |
| 4 | 0 | 1 (0.7) | 0.253* | 0 | 0 | NA |
| **Treatment (n, %)** |  |  |  |  |  |  |
| Milrinon | 11 (2.6) | 3 (2.1) | 1* | 0 | 0 | NA |
| Magnesium sulfate | 0 | 1 (0.7) | 0.253* | 0 | 0 | NA |
| IVIg | 39 (9.0) | 20 (13.7) | 0.109 | 0 | 0 | NA |
| **Outcome (n, %)** |  |  |  |  |  |  |
| Full recovery | 429 (99.5) | 142 (97.3) | 0.038* | 32 (100) | 176 (100) | NA |
| Recovery with complication or underlying diseases | 1 (0.2) | 3 (2.1) | 0.052* | 0 | 0 | NA |
| Death | 1 (0.2) | 1 (0.7) | 0.442 | 0 | 0 | NA |

(^): Mann- Whitney U test; (*): Fisher-exact test; other binary variables: Chi-square test, NA= Not applicable
