# Supplementary material for: Clinical, etiological and epidemiological investigations of hand, foot and mouth disease in southern Vietnam during 2015 – 2018
Source: PLoS Negl Trop Dis. 2020 Aug 17;14(8):e0008544. doi: 10.1371/journal.pntd.0008544 (PMC7451980; doi:10.1371/journal.pntd.0008544)
Supplement: S2 Table — (DOCX) [file pntd.0008544.s003.docx]

**Supplementary Table 2**: Frequency of other enterovirus serotypes detected in HFMD cases enrolled in the clinical study

| **Enterovirus serotypes** | **All patients** | **Inpatients** | **Outpatients** | **Severe patients** | **Mild patients** |
| --- | --- | --- | --- | --- | --- |
| Coxsackievirus A8 | 22 | 18 | 4 | 0 | 22 |
| Coxsackievirus A2 | 12 | 12 | 0 | 2 | 10 |
| Coxsackievirus A4 | 8 | 7 | 1 | 0 | 8 |
| Coxsackievirus A5 | 7 | 7 | 0 | 0 | 6 |
| Coxsackievirus A1 | 2 | 2 | 0 | 2 | 0 |
| Coxsackievirus B1 | 1 | 1 | 0 | 0 | 1 |
| Coxsackievirus B2 | 1 | 1 | 0 | 0 | 1 |
| Coxsackievirus B3 | 1 | 1 | 0 | 0 | 1 |
| Coxsackievirus B4 | 1 | 0 | 1 | 0 | 1 |
| Echovirus 18 | 0 | 4 | 0 | 0 | 4 |
| Echovirus 5 | 0 | 3 | 0 | 1 | 2 |
| Echovirus 6 | 0 | 0 | 1 | 0 | 1 |
| Echovirus 9 | 0 | 1 | 0 | 0 | 1 |
| Poliovirus 2 | 0 | 1 | 0 | 0 | 1 |
| Rhinovirus A | 0 | 1 | 0 | 0 | 1 |
