# Supplementary material for: Clinical, etiological and epidemiological investigations of hand, foot and mouth disease in southern Vietnam during 2015 – 2018
Source: PLoS Negl Trop Dis. 2020 Aug 17;14(8):e0008544. doi: 10.1371/journal.pntd.0008544 (PMC7451980; doi:10.1371/journal.pntd.0008544)
Supplement: S3 Table — (DOCX) [file pntd.0008544.s004.docx]

**Supplementary Table 3**: Comparing between infants and children

| **Demographics** | **Infants**  **N=234** | **Children**  **N= 962** | **P- value** |
| --- | --- | --- | --- |
| male/female | 147/87 | 599/363 | 0.875 |
| HCMC origin (n, %) | 105 (44.9) | 463 (48.1) | 0.371 |
| Day from onset to enrolment | 1 (0-10) | 1 (0-7) | 0.002* |
| Length of stay in the hospitals | 3 (1-16) | 4 (1-31) | 0.061* |
| Enviromental factors |  |  |  |
| Attend day care centers or school (n, %) | 8 (3.4) | 268 (27.9) | <0.001 |
| Number of siblings (median, range) | 0 (0-13) | 1 (0-8) | 0.372* |
| Exposure to HFMD patients (n, %) | 21 (9.0) | 129 (13.5) | 0.063 |
| **Clinical features** (n, %) |  |  |  |
| Mouth ulcers | 202 (86.3) | 860 (89.4) | 0.181 |
| Fever | 191 (81.6) | 745 (77.4) | 0.164 |
| Skin lesions | 195 (83.3) | 839 (87.2) | 0.120 |
| vesicles | 54 (23.1) | 259 (26.9) | 0.230 |
| papular | 72 (30.8) | 301 (31.3) | 0.878 |
| mix | 69 (29.5) | 279 (29.0) | 0.884 |
| Cough | 52 (22.2) | 239 (24.8) | 0.402 |
| Runny nose | 45 (19.3) | 192 (20) | 0.819 |
| Diarrhea | 36 (15.5) | 81 (8.4) | 0.001 |
| Drowsiness | 48 (20.6) | 215 (22.4) | 0.558 |
| Sweating | 2 (0.9) | 15 (1.6) | 0.414^ |
| Vomiting | 101 (43.2) | 424 (44.1) | 0.801 |
| Irritability | 62 (26.5) | 295 (30.7) | 0.211 |
| Myoclonus | 106 (46.9) | 462 (48.6) | 0.650 |
| Lethargy | 5 (2.1) | 22 (2.3) | 0.886 |
| Tremor | 9 (3.8) | 102 (10.6) | 0.001 |
| Ataxia | 0 | 3 (0.3) | 1^ |
| Nystagmus | 0 | 1 (0.1) | 1^ |
| Limb weakness | 2 (0.9) | 9 (0.9) | 1^ |
| Hypertension | 11 (4.7) | 58 (6.0) | 0.434 |
| **Results of blood tests, median (range)** |  |  |  |
| White blood cell (x1000) | 12.5 (2.5-35.9) | 12.2 (3.2-76) | 0.432* |
| Platelet (x1000) | 301 (29-849) | 325 (31-844) | 0.001* |
| Blood glucose (mg/L) | 101 (58-210) | 101 (42-233) | 0.908* |
| C reactive protein (mg/L) | 11.2 (0-103.4) | 9.16 (0-620) | 0.899* |
| **Highest grade** (n, %) |  |  |  |
| 1 | 38 (16.2) | 213 (22.1) | 0.047 |
| 2A | 161 (68.8) | 503 (52.3) | <0.001 |
| 2B1 | 10 (4.3) | 61 (6.3) | 0.230 |
| 2B2 | 3 (1.3) | 65 (6.8) | 0.001 |
| 3 | 21 (9.0) | 115 (12.0) | 0.198 |
| 4 | 1 (0.4) | 5 (0.5) | 1^ |
| **Treatment** (n, %) |  |  |  |
| Milrinon | 11 (5.7) | 58 (7.8) | 0.314 |
| Magnesium sulfate | 0 | 1 (0.1) | 1^ |
| IVIg | 25 (13.0) | 190 (25.7) | <0.001 |
| **Outcome** (n, %) |  |  |  |
| Full recovery | 232 (99.1) | 949 (98.6) | 0.540 |
| Recovery with complication or underlying diseases# | 1 (0.4) | 11 (1.1) | 0.479^ |
| Death | 1 (0.4) | 2 (0.2) | 0.480^ |
| The frequency of 4 common pathogens, (n, %) |  |  |  |
| EV-A71 | 39 (16.7) | 301 (31.3) | <0.001 |
| CV-A6 | 51 (21.8) | 145 (15.1) | 0.013 |
| CV-A10 | 30 (12.8) | 65 (6.8) | 0.002 |
| CV-A16 | 15 (6.4) | 122 (12.7) | 0.007 |

**Note to Supplementary Table X**: ^#^limb weakness and squint-eyed (n=2), limb weakness (n=5), “slow communication” (n=1), cranial nerve VII paralysis (n=1), unconsciousness (n=1), Guillain Barre syndrome (n=1), prolonged fever and paralyzed diaphragm (n=1), severe anemia (n=2) and subacute encephalitis (n=1). (*): Mann-Whitney U test; (^): Fisher-Exact test.
